# Plasma exchange following immunoglobulins for Guillain-Barré-Syndrome: a persisting problematic practice?

**DOI:** 10.3389/fneur.2025.1712202

**Published:** 2026-01-08

**Authors:** Guglielmo Lucchese

**Affiliations:** 1 Department of Neurology, University Medicine Greifswald, Greifswald, Germany; 2 Department of Experimental Medicine, University of Salento, Lecce, Italy; 3 Neurology Unit, “Vito Fazzi” Hospital, Lecce, Italy

**Keywords:** acute inflammatory demyelinating polyneuropathy (AIDP), acute motor axonal neuropathy (AMAN), intravenous immunoglobulin (IVIG), plasmapheresis (PP), treatment related fluctuation (TRF)

## Introduction

Current guidelines for treating GBS do not recommend combining the two available treatments, intravenous immunoglobulins (IVIG) and therapeutic plasma exchange (TPE), in any sequential order due to a lack of evidence of additional benefit (1–3). A reference work on GBS specifically recommends against TPE after IVIG (4). Nevertheless, combination therapy, mostly IVIG followed by TPE for difficult-to-treat GBS, is still reported (5–7). This practice has also been directly observed by and reported in personal communications to the author.

The question therefore arises: why do clinicians follow non-recommended procedures when confronted with less-than-optimal response to IVIG? And is there any rationale to support it? Here we try to find an answer to these questions in light of the current observational evidence, immunopathogenesis of the disease, and the mechanisms of action of the two therapeutic treatments.

## Pathophysiology of GBS

Guillain–Barré Syndrome (GBS) is an acute autoimmune polyradiculoneuropathy which often follows bacterial or viral infections (4). Molecular mimicry is the proven mechanism mediating autoimmunity after C. Jejuni infection in the axonal variants of GBS (acute motor axonal neuropathy, AMAN, and acute motor and sensory axonal neuropathy, AMSAN) and is a consequence of the structural similarity between the bacterial lipooligosaccharide and the GM1 and GD1a gangliosides present in the nodes of Ranvier and paranodal areas of peripheral nerves (8, 9). The pathogenesis of the demyelinating variant (acute inflammatory demyelinating polyradiculoneuropathy, AIDP) of GBS is less understood. Although some cases might be associated with anti-galactocerebroside autoantibodies, autoimmunity in AIDP appears to be driven by T-cell activation and cytokines like IFN-γ, TNF-α, and IL-6 (10–12).

Indeed, most patients suffering from GBS test negative for anti-ganglioside antibodies, especially in the clinical-electrophysiological AIDP variant, suggesting that other immune-mediated mechanisms are involved in GBS and that such alternative mechanisms are prevalent in the demyelinating variant (13). Recently, autoreactive CD4+ cytotoxic T helper 1 and CD8+ T cells targeting antigens of the peripheral nerve myelin sheath were detected in patients with AIDP (14). Nevertheless, anti-GM1 antibodies can also be found in AIDP (15). Moreover, both axonal and demyelinating subtypes of GBS can be mediated by complement-fixing anti-GM1 antibodies (16).

Binding of the C1q complement component to antibodies recognizing peripheral nerves activates the classical complement pathway on the nerve surface, thus driving neuroinflammation and nerve damage (17, 18). Evidence also suggests that classical complement activation underlies nerve injury in GBS regardless of axonal or demyelinating pathophysiology (17, 18).

The axonal vs. demyelination dichotomy in GBS diagnosis is supported by the electrophysiological study of nerve conduction, classically capable of distinguishing signs of axonal degeneration, like decreased distal compound muscle action potential (CMAP) amplitudes, from signs of damage to the myelin sheet, such as increased CMAP latency and temporal dispersion. Nevertheless, electrophysiology too might sometimes fail to be conclusive especially in the early phase of disease. Indeed, both demyelinating and axonal forms may manifest with nerve conduction blocks as shown by reduced ratio of proximal vs distal CMAPs. These blocks are usually longer lasting in AIDP whereas they tend to resolve quickly in anti-GM1 associated AMAN/AMSAN. The rapidly resolving conduction blocks in axonal GBS forma have therefore been named reversible conduction failure (RCF) and are considered a sign of nodal dysfunction.

## Current guidelines for GBS therapy

The previous paragraph briefly described the variability in the molecular mechanisms underlying a diagnosis of GBS. Two patients with the same diagnosis of GBS might be victims of either abnormal T-cell activation targeting myelin, predominant molecular-mimicry-elicited humoral autoimmunity damaging the nodal-paranodal portions of the nerve, or both.

Nevertheless, neither the clinical-electrophysiological aspects nor the immunopathogenetic mechanisms of the disease are of therapeutic relevance in clinical practice (1).

Currently, GBS therapy consists of intravenous immunoglobulins (IVIG) and therapeutic plasma exchange (TPE). Historically, TPE was the first treatment adopted (19). Subsequent RCTs comparing IVIG to TPE did not show evidence for superiority of one therapy over the other, and there are currently no identified subgroups of patients showing increased benefit from either TPE, IVIG, or TPE followed by IVIG (1). The European guidelines and the latest GBS reference work from The Lancet both argue that TPE after IVIG washes out the previous treatment, which is considered expensive and irrational (1, 4). Indeed, although direct evidence on IVIG removal by TPE is actually limited, immunoglobulins are likely to be significantly removed by plasma exchange (20).

## Pharmacodynamics of IVIG

IVIG comprises about 90% IgG and exerts immunomodulatory functions through a variety of mechanisms (21). It can bind and neutralize circulating autoantibodies, the anaphylatoxins C3a and C5a, and other complement proteins. At high doses, IgG in IVIG saturates activating Fcγ receptors (FcγRs) on immune cells, thereby blocking their activation and reducing immune complex-driven inflammation and autoimmunity. IVIG also suppresses the activation of monocytes and macrophages and interferes with the maturation and differentiation of dendritic cells. In addition, it promotes the release of anti-inflammatory cytokines by innate immune cells and exerts cytotoxic effects on neutrophils and eosinophils. IVIG can trigger apoptosis in dendritic cells as well as effector Th1 and Th17 cells, while enhancing immunosuppressive pathways through expansion of regulatory T cells. Furthermore, it inhibits B-lymphocyte proliferation, thereby modulating antibody production. Arumugham and Rayi provide a detailed overview of all these mechanisms (21). In sum, IVIG exerts a modulating effect on innate, humoral, and cell-mediated immunity, contributing to its clinical efficacy.

## Mechanism of action of TPE

TPE exerts its effect by removing circulating antibodies, immune complexes, complement factors, cytokines, and other pro-inflammatory molecules (17). At the same time, the treatment may also modulate cellular immunity by altering the ratio of T helper type-1 (Th1) and type-2 (Th2) cells in peripheral blood, suggesting that TPE efficacy in dysimmune diseases goes beyond the mere mechanical removal of autoantibodies (22, 23).

## Arguments for TPE after IVIG

The main justification for TPE after ineffective IVIG treatment is the lack of alternatives. When a patient's clinical condition deteriorates after timely IVIG administration, the clinician is left with the dilemma of waiting for spontaneous recovery or switching therapy. A possible third option, repeating IVIG therapy, was recently ruled out by an RCT showing that a second cycle of immunoglobulins in GBS patients with poor prognosis is not only useless but also potentially detrimental (24, 25).

TPE after IVIG might find additional theoretical support in the notion that AMAN appears to respond suboptimally to IVIG, and therefore some authors still advocate for switching treatment from IVIG to TPE in selected GBS cases, such as AMAN subtypes (5, 26). Following this argument, one might wonder why start the patient on IVIG in the first place.

## Arguments against TPE after IVIG

Combination therapy is currently not indicated by any guidelines, with the exception of one isolated mention to consider the procedure in the 2020 European pediatric guidelines (1–3, 27). The main reason for not recommending the IVIG-to-TPE switch is that no available evidence suggests such a practice is useful (28). Moreover, quickly washing out IVIG may be undesirable. Exposing patients to both treatments in such a sequential order might not only expose them to the adverse events of both without any expectation of benefit, but also be counterproductive. Indeed, combining IVIG and TPE is associated with higher mortality (29), although it is expected that patients undergoing both treatments are also more severely affected by the disease.

There are additional caveats to switching from IVIG to TPE. First, it is important to differentiate between failed response to treatment and treatment-related fluctuations (TRF). TRF is defined as “one or more deteriorations after initial improvement or stabilization after treatment (plasma exchange or immunoglobulins)” and may occur days after disease onset and therapy initiation (30, 31). The key elements is distinguishing improvement or stability of the clinical course, even for just a few days after the beginning of therapy, from progressive deterioration. At present, a TRF is an indication to repeat first-line treatment, either IVIG or TPE, under the assumption that temporary improvement (or stabilization) of clinical course is sign of partial treatment efficacy, albeit insufficient in the context of ongoing autoimmune damage underlying the treatment-related-fluctuations (1, 23). Switching to TPE from IVIG in the case of a fluctuation is not only not recommended but also irrational.

Second, although GBS is classically considered a post-infectious disease, para-infectious cases may occurr (32–34). In such cases, TPE requires additional considerations. TPE might be used for treating viral infections, mainly by removing cytokines and abnormal coagulation factors (35). However, one has to consider that the procedure might also remove critically important neutralizing antibodies, whereas IVIG appears to be beneficial for both autoimmune and infectious diseases (20, 36, 37). TPE after IVIG should therefore be very critically evaluated in para-infectious GBS.

## Alternatives to a problematic practice

Should we treat patients with GBS with TPE after lack of response to IVIG? We should not, in light of the evidence currently available. One possible major reason explaining why TPE is considered after IVIG in some cases of GBS despite lack of evidence, may be the frustration due to the lack of improvement or further decline of respiratory and motor functions. In such cases one must consider (and explain to the patients and relatives) that the immune-mediated attack on the peripheral nerves can result in axonal loss with classic Wallerian degeneration. This axonal degeneration may occur as the primary pathological process or as a secondary consequence. Importantly, wallerian degeneration is highly unlikely to reverse over the course of days to weeks, and therefore supportive care is of paramount importance in this phase, whereas a second line treatment like TPE after IVIG is unlikely to yield beneficial effects.

Do we need RCTs on plasma exchange after IVIG to definitively answer the question? Possibly. May we choose between TPE and IVIG as first-line therapy according to electrophysiological variant? The opinion of the author is that this might be a reasonable option if it does not delay treatment in any way. This is currently not the case given the difficulty to univocally differentiate axonal from demyelinating forms electrophysiologically due for instance to phenomena like RCF in AMAN/AMSAN. Indeed, even though exactly the RCF cases of AMAN, with or without ganglioside antibodies, are the ones that are most likely to maybe respond more promptly to TPE compared to IVIG (38), it is currently practically impossible to identify them with certainty in the first few days of the disease. Testing for anti-ganglioside antibodies is of no more help, because of the long turn-around time and not conclusive pathognomonic value. For all these reasons, the most recent European guidelines do not support the clinical distinction between axonal and demyelinating GBS. Nevertheless, a very recent publication shows promising data on the associations of the two biomarkers peripheral and periaxin with axonal and myelin damage, respectively, thus suggesting a true pathological difference (39). Further research is needed to assess if these biomarkers have a place in daily clinical practice.

Do we need more studies on specific treatment targeting specific pathogenetic mechanisms the disease? Definitely yes. Newer approaches to GBS treatment are showing remarkable efficacy including a complement C1q inhibitor, ANX005, and imlifidase (40, 41). Possibly, these approaches will obviate the need for a trial of TPE after IVIG.
